# Spatial organization of olfactory receptor gene choice in the complete V1R-related ORA family of zebrafish

**DOI:** 10.1038/s41598-022-17900-x

**Published:** 2022-08-31

**Authors:** Daniel Kowatschew, Shahrzad Bozorg Nia, Shahzaib Hassan, Jana Ustinova, Franco Weth, Sigrun I. Korsching

**Affiliations:** 1 Institute of Genetics, Mathematical-Natural Sciences Faculty of the University at Cologne, Zülpicher Str. 47A, 50674 Cologne, Germany; 2 Zoological Institute, Department of Cell- and Neurobiology, Karlsruhe Institute of Technology, Fritz-Haber-Weg 4, 76131 Karlsruhe, Germany

**Keywords:** Cell biology, Evolution, Genetics, Neuroscience

## Abstract

The vertebrate sense of smell employs four main receptor families for detection of odors, among them the V1R/ORA family, which is unusually small and highly conserved in teleost fish. Zebrafish possess just seven ORA receptors, enabling a comprehensive analysis of the expression patterns of the entire family. The olfactory organ of zebrafish is representative for teleosts, cup-shaped, with lamella covered with sensory epithelium protruding into the cup from a median raphe. We have performed quantitative in situ hybridization on complete series of horizontal cryostat sections of adult zebrafish olfactory organ, and have analysed the location of *ora*-expressing cells in three dimensions, radial diameter, laminar height, and height-within-the-organ. We report broadly overlapping, but distinctly different distributions for all *ora* genes, even for *ora3a* and *ora3b*, the most recent gene duplication. Preferred positions in different dimensions are independent of each other. This spatial logic is very similar to previous reports for the much larger families of odorant receptor (*or*) and V2R-related *olfC* genes in zebrafish. Preferred positions for *ora* genes tend to be more central and more apical than those we observed for these other two families, consistent with expression in non-canonical sensory neuron types.

## Introduction

To detect a multitude of odors, the sense of smell employs a complex array of receptor molecules organized into four major and some minor receptor gene families, which together can represent up to one tenth of the total genome in some mammalian species^1^. The same four families—odorant receptors proper (ORs), two types of 'vomeronasal' receptors (V1R-related ORAs and V2R-related OlfCs), and the trace amine-associated receptors (TAARs)—are present in teleost fish^2^. Note that a distinction commonly made for mammalian receptors into olfactory receptors proper (ORs and TAARs) and pheromone receptors (V1Rs and V2Rs) is not applicable to non-tetrapod species, who do not possess the segregated vomeronasal organ eponymous for the VRs, and whose 'division of labor' between receptor families is quite different^2^. Teleost olfactory receptor repertoires tend to be much smaller than those of tetrapods, and even the relatively large olfactory receptor repertoire of zebrafish amounts only to 350 genes in total^2^. In particular one major family, the V1R-related ORA receptors, only consists of five to eight receptors in teleosts^3,4^, allowing comprehensive studies encompassing the entire ORA family. A hallmark of olfactory receptor gene expression is the monogenic expression (one neuron-one receptor)^5^, coupled with a not-quite-random expression pattern, in which cells expressing the same receptor are scattered within the sensory surface, but nevertheless show preferred locations amounting to distinctly different 'centers-of-gravity' for different genes. Such patterns have been observed and sometimes quantified for olfactory receptors from different families, usually restricted to observations of a single spatial parameter^6–11^. Recently a three-dimensional analysis of spatial expression patterns was performed for some members of another receptor family, V2R/OlfCs^12^. In zebrafish, the OR and OlfC families encompass 150 and 60 genes, respectively, a challenging number for a comprehensive analysis. In contrast, the V1R-related ORA gene family only consists of seven members, which allows to determine the spatial expression patterns of the entire family. We have recently developed a thorough analysis method to quantify and compare three-dimensional spatial distribution patterns of olfactory receptor-expressing neurons^13^. Here we use this method for a comprehensive determination of spatial expression patterns for all seven *ora* genes. Using in situ hybridization we have quantified cell position in three dimensions. We observe broadly overlapping, but distinctly different expression zones for different ORA receptors spanning nearly the full range of the sensory surface. This spatial logic thus constitutes a general feature shared by zebrafish ORA, V2R/OlfC and OR receptor families.

## Results

### Comprehensive analysis of spatial expression patterns for a complete olfactory receptor family

**Figure 1:**
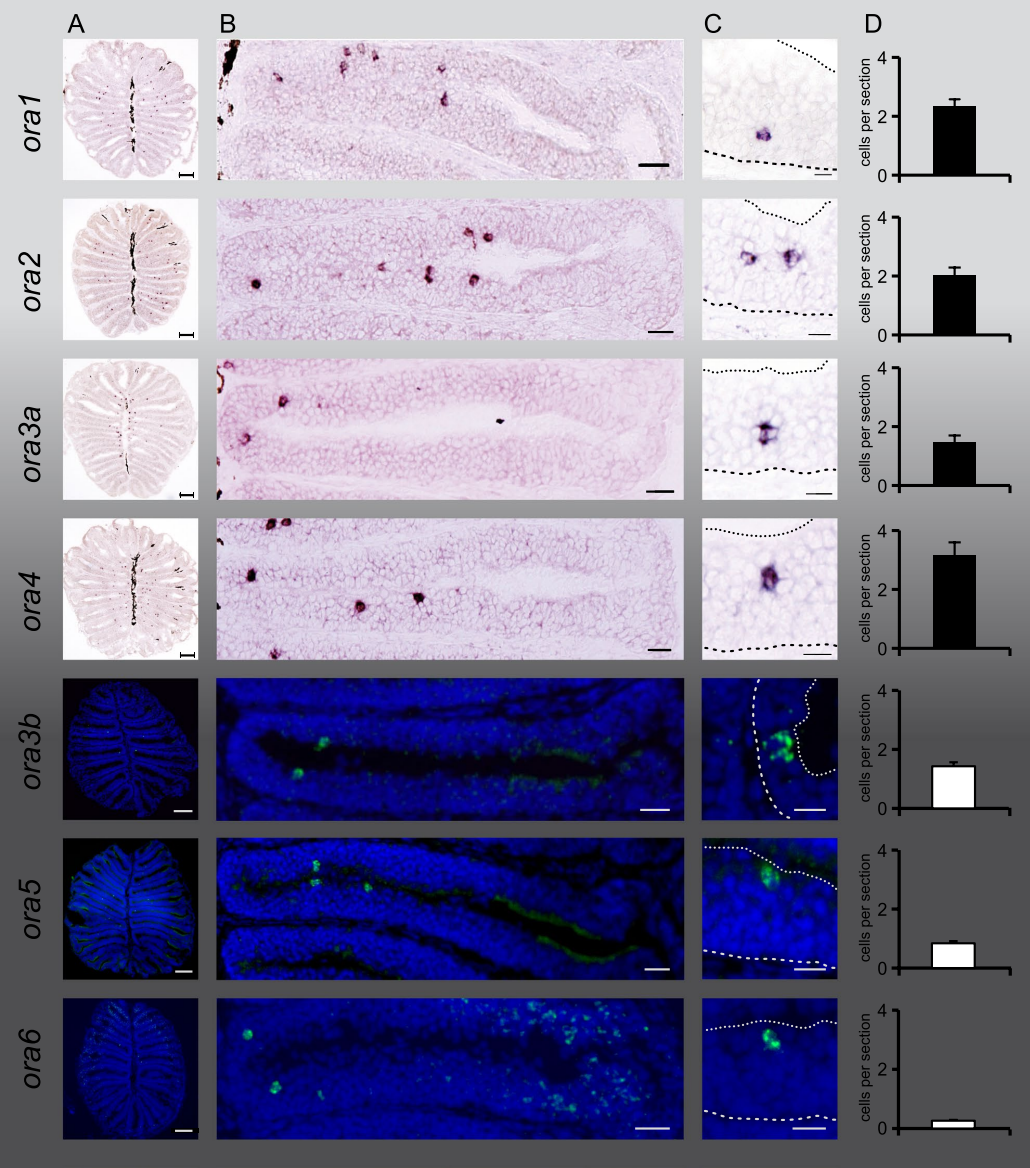
Expression of all seven *ora* genes in sparsely distributed cells in the olfactory epithelium. Horizontal sections of adult zebrafish olfactory epithelia were hybridized with probes for all seven ora genes, as indicated. Chromogenic detection by NBT/BCIP for *ora1, ora2, ora3a* and *ora4*, fluorigenic detection with TSA for *ora3b, ora5*, and *ora6*. Column (**A**) shows representative complete sections labeled with the respective probes. Column (**B**), higher magnifications of a single lamella from different sections. All panels have the same orientation, center (median raphe) to the left and periphery to the right. Note that for better visualization sections with above average frequency were selected, thus values are higher than the average values shown in column (**D**). Column (**C**), higher magnification shows positions of single cells within the lamella. Coarse dashed lines depict the border to the basal lamina and fine dashed lines depict the apical border to the lumen, cf. Fig. 2. (**A**) Scale bars correspond to 100 µm, (**B**) scale bars 20 µm (30 µm for ORA1), (**C**) scale bars 10 μm. Column (**D**), bar graphs show number of labeled cells per section for each *ora* gene (mean +/− SEM, for number of sections analysed see SI Fig. 1).

**Figure 2:**
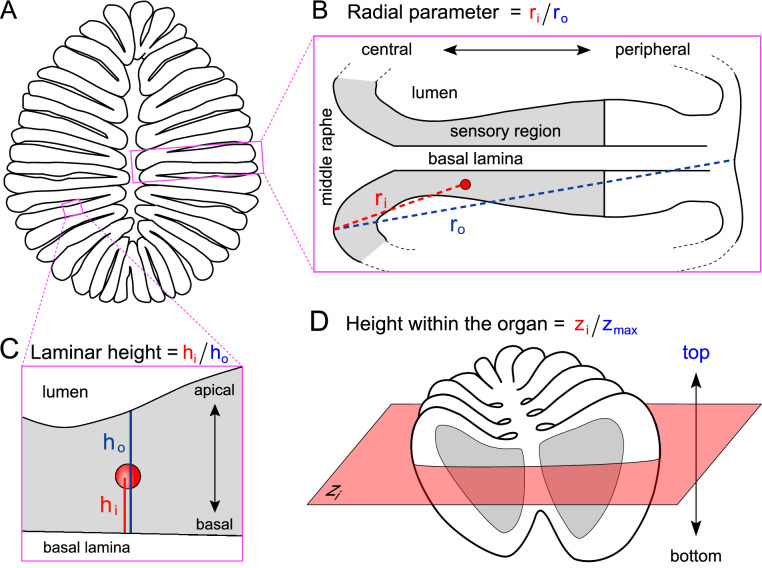
Schematic representation of spatial coordinates measured. (**A**) Horizontal cross section of an olfactory epithelium. (**B**) Enlargement shows the radial parameter (r_i_), measured from middle raphe between lamellae to center of labeled cell and normalized to maximal radius (r_o_, including non-sensory area). One lamella is shown. (**C**) Enlargement shows the laminar height parameter (h_i_), measured from the border between basal lamina and the sensory layer to center of labeled cell and normalized to maximal height (h_o_, measured at position of labeled cell, as laminar height varies throughout the lamella). (**D**) The complete olfactory organ, all three spatial coordinates are shown. height-within-the-organ is measured as section number z, beginning with the most basal section still containing sensory surface (bottom to top).

The *v1r/ora* gene family is the only one of the four major olfactory receptor families (OR, TAAR, V1R/ORA, V2R/OlfC), which has a very limited size in teleost fish. In zebrafish seven genes comprise the entire family allowing us to perform an in depth investigation of the spatial pattern of *ora*-expressing sensory neurons for each *ora* gene. We performed in situ hybridization with probes for all *ora* genes on cryostat sections of the olfactory epithelium of zebrafish to obtain a three-dimensional spatial representation of *ora* gene expression. For each gene, complete series of sections were obtained for 5–15 olfactory epithelia of adult fish. Chromogenic detection of hybridized probes was sufficient for analysis of expression for *ora1, ora2, ora3a* and *ora4*, but for *ora3b, ora5* and *ora6* it was necessary to use a more sensitive fluorigenic detection method based on tyramide signal amplification (TSA, see “Materials and methods” section) due to the low expression of these receptors (Fig. 1). The TSA method yielded approximately threefold higher frequency of expression than the chromogenic detection (NBT/BCIP) method (SI Fig. 1). Patterns of labeled cells were indistinguishable between chromogenic and fluorigenic detection. The position of labeled cells was determined as distance from the center of the organ (radial parameter), distance from the basal lamina of the sensory epithelium (laminar height) and distance from the bottom of the olfactory organ (height-within-the-organ), resulting in a three-dimensional representation (Fig. 2). All three parameters were normalized to their respective maximal values (Fig. 2), for details see “Materials and methods” section.

### Three expression domains are distinguishable in quantitative analysis of radial distribution, each populated by more than one gene

**Figure 3:**
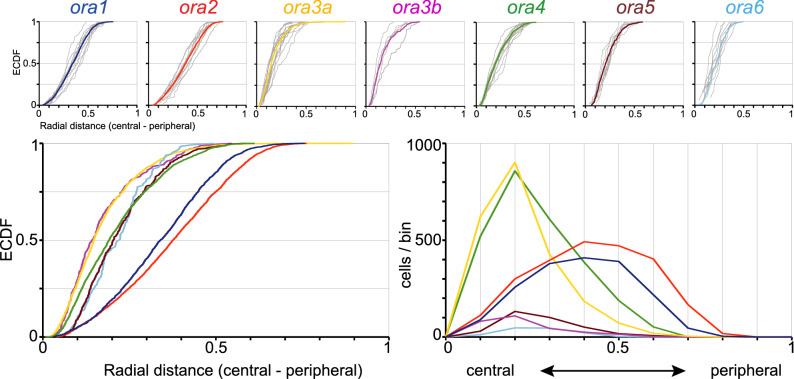
Quantitative analysis of radial position shows three subgroups of *ora* genes. Radial positions of *ora*-expressing cells were quantified for all seven *ora* genes. Complete series of sections from five to fifteen olfactory epithelia were evaluated for each *ora* gene. Radial position within the section was normalized to maximal radius, i.e., length of the lamella containing the respective labeled cell. Upper row: The resulting distributions of relative radius (from 0, innermost to 1, outermost) are shown unbinned (ECDF). Light grey curves represent the distribution for individual olfactory organs, colored curves represent the cumulative distribution for the respective genes. The color code for the *ora* genes is the same as in later figures to facilitate comparisons between different positional parameters. Lower row: Overlay of the seven distributions shown individually in upper row, both as ECDF (left panel) and histogram (right panel). Different genes exhibit different centers-of-gravity (mean) and different skewness. Note a segregation in three groups, with ORA3a and ORA3b in a central group, ORA1 and ORA2 in a peripheral group and the remaining three receptors in an intermediate group.

The distribution of radial positions of *ora*-expressing cells was quantified for all seven *ora* genes. Complete series of sections from five to fifteen olfactory epithelia were evaluated for each *ora* gene. Radial position was measured from the apex of the lamellar ‘curve’, i.e., closest to the median raphe, to the cell soma center, and normalized to the distance between this apex position (most central) and the border of the epithelial section (most peripheral). Results are shown as histogram and as empirical cumulative distribution function (ECDF), the latter being an unbinned representation with correspondingly higher resolution (Fig. 3). While all distributions show broad overlap, three subgroups with distinctly different centers-of-gravity become apparent, one of them formed by ORA1 and ORA2, another by ORA3a/3b, and the third by ORA4,5,6. ORA1 and ORA2 are robustly more peripheral than all other distributions, which cluster together at more central positions. The maximal vertical distances between the distributions for ORA1, ORA2 and the other five ORAs are large and range between 0.37 and 0.55 (SI Table 1). Within the more central group of receptors a further subdivision into ORA3a/3b and ORA4,5,6 is apparent. ORA3a/3b occupy the most central positions and are extremely similar to each other (Fig. 3). Between these two groups maximal vertical distances still range between 0.19 and 0.31 (SI Table 1). In all pairwise comparisons genes from one of the three subgroups are significantly different from all genes of the other two subgroups (SI Table 1). Taken together, analysis of radial position shows three significantly different, albeit broadly overlapping expression domains, each populated by two to three different *ora* genes.

### Analysis of laminar height delineates further subdivisions between genes

**Figure 4:**
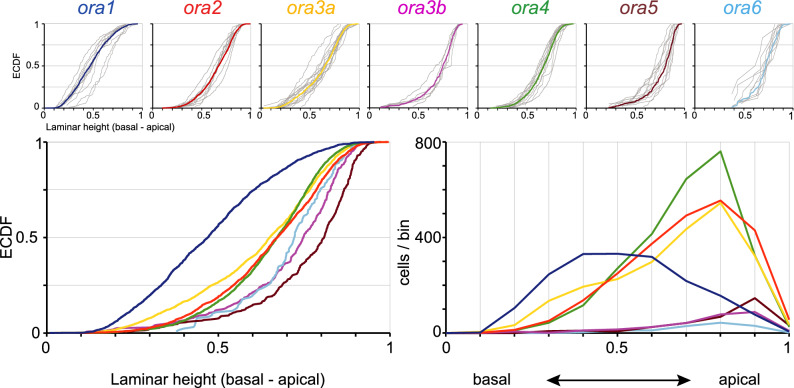
Quantitative assessment of laminar height distributions of *ora*-expressing neurons. Laminar height of *ora*-expressing cells was quantified for all seven *ora* genes. Complete series of sections from five to fifteen olfactory epithelia were evaluated for each *ora* gene. Height within the lamina was normalized to maximal laminar thickness at the position of the cell. Upper row: The resulting distributions of relative laminar height (from 0, most basal to 1, most apical, i.e., bordering to the lumen) are shown unbinned (ECDF). Light grey curves represent the distribution for individual olfactory organs, colored curves represent the cumulative distribution for the respective genes. The color code for the *ora* genes is the same as in other figures to facilitate comparisons between different positional parameters. Lower row: Overlay of the seven distributions shown individually in upper row, both as ECDF (left panel) and histogram (right panel). Different genes exhibit different centers-of-gravity (mean) and different skewness.

Next, we analysed the preferred positions of *ora-*expressing cells in terms of laminar height, i.e., distance from the basal lamina underlying the sensory epithelium. Height within the lamina was normalized to maximal laminar thickness. Laminar height was quantified for all seven *ora* genes, using complete series of sections from five to fifteen olfactory epithelia per gene. Results are shown as histogram and as empirical cumulative distribution function (ECDF) (Fig. 4). Nearly all distributions are significantly different from each other (SI Table 1). Maximal vertical distance between distributions reaches 0.63 for the ORA1/ORA5 comparison. Notably the receptor pairs with similar (ORA1/ORA2) or nearly identical (ORA3a/ORA3b) radial distribution (Fig. 3) are clearly distinct in terms of their laminar height distribution, with ORA2 more apically centered compared to ORA1 and ORA3b with more apical preference compared to ORA3a (Fig. 4). Even the third radial subgroup comprised of ORA4, 5, 6 segregates to some extent in their laminar height distribution (Figs. 3, 4). In other words, preferred radial and preferred basal/apical position are not correlated and thus appear to be determined independently.

### The third spatial parameter analysed, height-within-the-organ, divides *ora* genes in yet other subgroups

**Figure 5:**
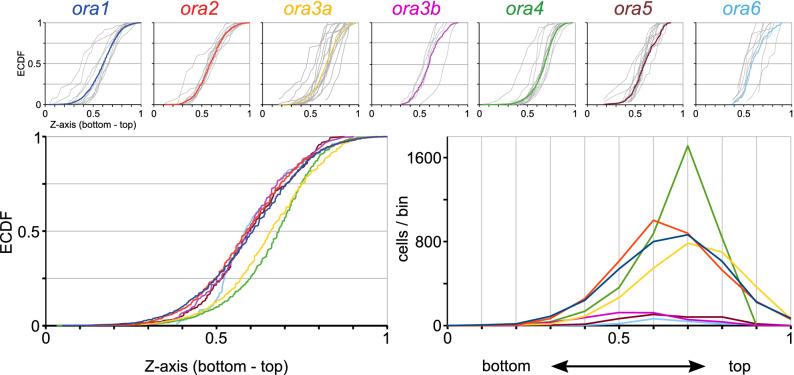
*ora4*-expressing cells show preferential expression in the top of the olfactory organ. Quantitative assessment of distribution of ora-expressing neurons along the vertical z-axis (height-within-the-organ). Height within the olfactory organ was quantified as section number in a series of horizontal sections, and normalized to the total number of sections containing sensory epithelium, using the same set of cells, for which laminar height was determined. Relative height-within-the-organ ranges from 0 (bottommost section) to 1 (top section, near to the opening of the bowl-shaped olfactory organ). Upper row: The resulting distributions are shown unbinned (ECDF). Light grey curves represent the distribution for individual olfactory organs, colored curves represent the cumulative distribution for the respective genes. The color code for the *ora* genes is the same as in other figures to facilitate comparisons between different positional parameters. Lower row: Overlay of the distributions shown individually in the upper row, both as ECDF (left panel) and histogram (right panel). Due to lower resolution (all cells within one 10 µm section are assigned to a single z value) the ECDF curves appear ragged.

Finally we wished to investigate whether height within the olfactory organ (z axis) would be differently distributed for different *ora* genes. Height-within-the-organ was quantified as section number in a series of horizontal sections, and normalized to the total number of sections containing sensory epithelium, ranging from 0 (bottommost section still containing sensory epithelium) to 1 (top section, near to the opening of the bowl-shaped olfactory organ). We report that distributions are somewhat more similar than those found for radius and laminar height coordinate, with maximal vertical distances in the range of 0.06–0.31 (SI Table 1). There are two subgroups apparent, with ORA3a and ORA4 occupying positions closer to the lumen of the olfactory organ (top), whereas the other five genes tend to be expressed somewhat closer to the base of the olfactory organ (bottom) (Fig. 5, SI Fig. 2). All pairwise comparisons between subgroups show highly significant differences (SI Table 1). These subgroups are different from those observed for the other two spatial parameters, e.g. ORA4 groups with ORA5 and ORA6 for the radial parameter, with ORA2 and ORA3a for laminar height, and only with ORA3a for the z axis (Figs. 3, 4, 5, SI Fig. 2). Thus, analysis of the third spatial parameter extends the conclusion drawn from the comparison of radius and laminar height: all three spatial parameters appear independently regulated.

**Figure 6:**
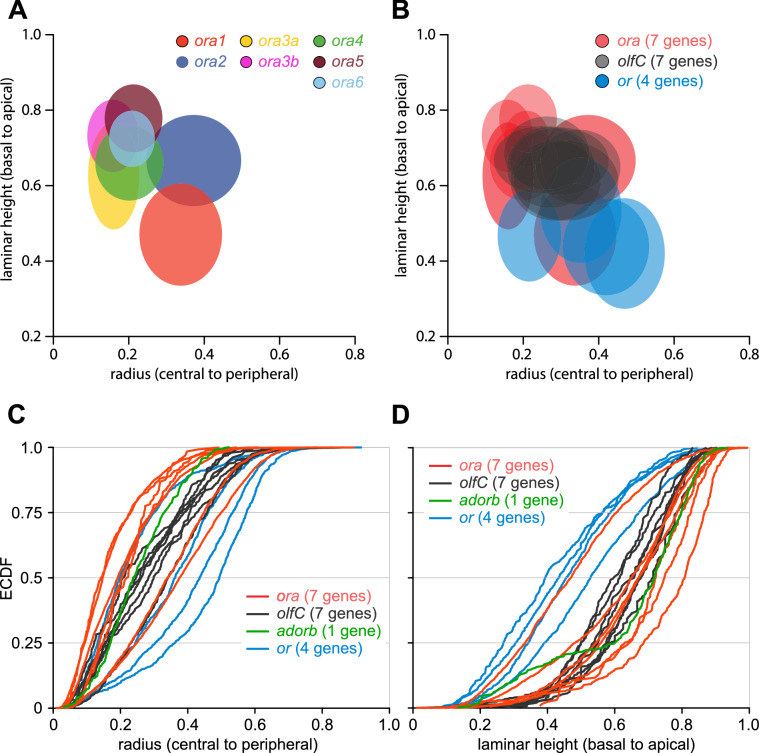
Multidimensional analysis of spatial expression patterns shows significantly different expression zones for all ora genes and clear segregation from two other olfactory receptor families. (**A**) Comparison of spatial distribution parameters between *ora* genes. Schematic representation of spatial distributions for all *ora* genes by ellipses ranging from the 1st to the 3rd quartile value for radial (x axis) and laminar height (y axis) parameter. Color code for *ora* genes as before. (**B**) Comparison of distributions for *ora* versus *olfC* and odorant receptor (*or*) genes using the same coordinates and representation as in (**A**). Note that the color code is changed compared to panel (**A**). The raw data for *olfC* genes are taken from^11^, raw data for the radial parameter of *or* genes are from^7^. For names and quartile values of *or* and *olfC* genes see SI Table 1. (**C**,**D**) Distributions of *ora*-expressing OSNs intercalate between those of other olfactory receptor families. Distributions are shown as ECDFs. Red lines, *ora* genes; black, *olfC* genes; blue, *or* genes. Panel (**C**) shows the distribution for the radial coordinate, panel (**D**) depicts the laminar height parameter.

Taken together, the three-dimensional distributions for each of the seven *ora* genes are significantly different from each other in at least one and up to three dimensions (Fig. 6, SI Fig. 2, SI Table 1).

### Evolutionary divergence times do not correlate with extent of spatial pattern differences

Of all seven zebrafish *ora* genes o*ra3a* and *ora3b* are most closely related to each other and in fact lie closely adjacent in the genome (4 kb distance, see SI Table 1). We investigated the origin of this local gene duplication by performing tblastn searches in all available WGS databases of Cypriniformes and neighboring orders (Characiformes, Siluriformes, and Clupeiformes). We observe that zebrafish o*ra3a* and *ora3b* genes possess direct orthologs in many suborders and families of Cypriniformes (e.g. loaches, sucker, minnows, carps) but not in the adjacent orders (Clupeiformes [e.g. herring], Siluriformes [e.g. catfish], Characiformes [e.g. piranha]) (SI Fig. 3). This suggests that *ora3a* and *ora3b* genes arose from a local gene duplication of an ancestral *ora3* gene in the common ancestor of Cypriniformes, i.e., about 100 million years ago (mya)^14^. This period of time has been sufficient to diversify the specification mechanism for laminar height and z-axis, but not that for the radial parameter. The divergence times for ORA3 *vs.* the ORA4 clade are considerably larger: both clades are present in bony and cartilaginous, but not in jawless fish, so the birth of these clades can be dated to about 470 million years ago (mya)^14^. Nevertheless, only some distribution differences (radius and height-within-the-organ) are larger for ORA3a,b *vs.* ORA4 than for ORA3a *vs*. ORA3b (Fig. 6, SI Table 1). The overall largest distribution differences are observed between ORA2 and ORA3a,b (radial parameter), and between ORA1 and ORA5 (laminar height), i.e., between the three ancestral clades ORA1/2, ORA3/4 and ORA5/6. Since all three ancestral clades are already present in jawless fish^15^ this amounts to about 600 mya divergent evolution^14^. But again, some distribution differences between the ORA1,2 and ORA3,4 and ORA5,6 clades are very small, consistent with the observed lack of correlation between spatial parameters. Thus, there is no general tendency for distributions of different genes to segregate increasingly with evolutionary age.

### Spatial relationships between different olfactory receptor families

Next we wished to compare, how the spatial organization of *ora* gene choice described above compares to that of other olfactory receptor families of zebrafish, for which such data are available. A recent study has performed the same three-dimensional analysis of spatial expression patterns for seven members of the V2R-related OlfC family^12^. We integrated the raw data from this study in our combined representation of the distributions for all *ora* genes to compare the two families directly (Fig. 6). Note that individual distributions from these two families are similarly broad as measured by half-width, albeit those for the *ora* genes tend to be somewhat narrower (SI Table 1). However, for both the radial and the laminar height parameter the distributions of the *ora* genes are much more divergent than those of the *olfC* genes, and in fact the range of median values observed for *ora* genes encompasses the much narrower range for the *olfC* distributions. For the height-within-the-organ parameter the distributions of *ora* genes lie closer to the top of the organ than the olfC distributions (SI Fig. 2, SI Table 1). Taking all three parameters into account, all *ora* genes show significantly different distributions to all of the *olfC* genes: even *ora1*, which is very similar to one of the *olfC* genes for the radial parameter, is very different in laminar height (SI Table 1). The general principle of broadly overlapping, but distinctly and significantly different distributions thus holds for *ora* and *olfc* genes combined.

We then compared the distribution of *or* genes in all three dimensions with that of the *ora* genes described here. For the radial and height-within-the-organ parameters we integrated the raw data from earlier studies, which analysed four *or* genes^7,16^. For the same four genes we now also determined the distributions for laminar height. We note that for radial and laminar height parameter the median values for the distributions of the *or* genes are similarly divergent as for the *ora* genes, and, like those, much more divergent than the closely clustering *olfC* genes (Fig. 6). For the radial parameter, all four *or* genes show significantly different distributions from each other, for the laminar height parameter the majority of comparisons shows significant differences (SI Table 1). Interestingly, the range of the distributions is characteristically different for *or* and *ora* genes. With very few exceptions, the centers-of-gravity tend to lie more peripheral (radial parameter) and more basal (laminar height parameter) for the *or* genes compared to the *ora* genes (Fig. 6). There is a single *or* gene, whose radial distribution (but not laminar height or height-within-the-organ) is very similar to some *ora* genes (*ora4, 5, 6*) and a single *ora* gene (*ora1*), whose laminar height distribution (but not radius or height-within-the-organ) is very similar to some *or* genes (Fig. 6, SI Table 1). Distributions for height-within-the-organ are much more divergent for the *or* genes than for the ORA family (SI Fig. 2). Interestingly, height-within-the-organ appears anti-correlated with radius for *or* genes (SI Fig. 2), the only noticeable correlation between parameters in all three gene families.

Finally we integrated the raw data from our previous study of a single gene 'family' of *adorb* olfactory receptors^17^. This receptor (synonym A2c) was recently detected as an olfactory receptor for nucleotides^18^. Despite being an atypical olfactory receptor with very minor evolutionary dynamics^17,18^, the *adorb* receptor follows the same pattern of spatial distribution as the larger olfactory receptor families (Fig. 6).

Taken together, genes from all olfactory receptor families analysed (three major and one minor family) exhibit the same spatial patterns: broadly overlapping distributions with characteristically different centers-of-gravity for cells expressing different olfactory receptor genes.

## Discussion

Here we have performed a quantitative analysis of spatial expression patterns for an entire family of olfactory receptor genes, the *ora* genes, using the vertebrate model organism zebrafish. We find distinctly different spatial patterns for cells expressing the different *ora* genes, notwithstanding the broad overlap these distributions exhibit. In fact, each of the seven *ora* genes – which constitute the entire ORA family in zebrafish—exhibits a spatial pattern significantly different from all others whenever all three dimensions analysed (radius, laminar height, height-within-the-organ) are taken into account. We observe that preferred positions are not correlated across dimensions. An example of this lack of correlation is found with the two closely related genes *ora3a* and *ora3b,* which are nearly identical in their radial distribution, but distinctly and significantly different in two other dimensions (laminar height and height-within-the-organ).

The zebrafish olfactory epithelium contains several different types of olfactory sensory neurons, which show different laminar height preferences for their somata. Of the two major populations, OMP-positive ciliated neurons are located basal to microvillous neurons^13^. There are currently three minor populations known, of which crypt neurons are the best characterized. ORA4 is the only known olfactory receptor expressed in crypt neurons and seems to be expressed in the entire population^19^. Crypt neuron markers S100 and TrkA immunoreactivity^19,20^ show cells in very apical positions within the lamellae, above ciliated and microvillous neurons^13,21^, i.e., at large laminar height, which is consistent with the topology of *ora4*-expressing cells reported here. The cell types expressing the other ORA receptors are not known. ORA1 shows the most basal distribution, and is ancestral to all mammalian V1Rs^3^, which are expressed in microvillous neurons. Thus ORA1 conceivably might be expressed in microvillous neurons. On the other hand three receptors (ORA3b, ORA5, ORA6) possess an even more apical distribution than ORA4 (visible as right shift of the corresponding ECDF curves), which would be consistent with the location of another minor cell type, kappe neurons, for whom no receptor has been identified so far^13^. Either kappe or potentially other, so far unknown minor OSN types could be candidates for expression of ORA3b, ORA5, and ORA6. But note that the distributions measured for ciliated and microvillous neuronal markers^13,21^ average over many different receptors, and distributions for individual receptors might deviate considerably from the values averaged over the entire receptor family. The third minor cell population, pear neurons, is also located very apically, but has been shown to express the *adorb* (synonym A2c^18^) gene—an olfactory receptor for adenosine—and is therefore not expected to harbor additional olfactory receptor genes^17,18^.

Zebrafish, like other fish, possess a single olfactory epithelium, in which all the different olfactory receptor families are expressed. This is in contrast to the mammalian olfactory system, which segregates vomeronasal receptor families in a separate vomeronasal epithelium and only keeps ORs and TAARs in the main olfactory epithelium. Thus in the fish olfactory system the question arises how the expression of these different receptor families is arranged relative to each other. Previously it had been shown that individual distributions for the V2R-related OlfC family^12^ are similarly broad as observed for the odorant receptor family^7^. Nevertheless, the centers-of-gravity for these two families were found to be clearly, different, with the relatively narrow range of values observed for the V2R-related OlfC family^12^ intercalated within the broader range observed for the odorant receptor family^7^. Here we report that the range of distributions observed for the V1R-related *ora* genes is similarly broad as that of the *or* genes, with which they intercalate to some extent, albeit the ORA distributions tend to lie more central than the OR distributions (Fig. 6). For the second parameter, laminar height, the difference between ORA and OR distributions is more extreme, with all but one ORA distribution clearly more apical than all OR distributions analysed (Fig. 6).

Furthermore, the range of centers-of-gravity for the ORA distributions encompasses the narrow range observed for the distributions from the OlfC family^12^. *or* genes and *olfC* genes are known to be expressed in ciliated and microvillous neurons, respectively^22^. The characteristic differences of *ora* gene distributions compared to both *or* and *olfC* genes are consistent with the hypothesis discussed above that *ora* genes are expressed in other cell populations beyond ciliated and microvillous neurons, with the possible exception of ORA1, whose distribution lies in the midst of those observed for the ORs, and for one of the three parameters (radius) is also very similar to one of the *OlfC* genes.

The topology common to all these olfactory receptor genes consists of a 'half-random' distribution of cells, with individual distributions possessing unique 'centers-of-gravity' but nevertheless overlapping extensively with those from other genes due to their broad nature. In particular patterns from one gene family are intercalated with those from other families. Even the single gene 'family' of *adorb* olfactory receptors (synonym A2c^18^) follows this pattern (this paper and^17^, [SI figure 6]).

The initial observations for rodent odorant receptors seemed to imply a much sharper delineation between distributions of cells expressing different ORs and two to four different expression zones with little overlap between them have been proposed for rodent^23,24^ and primate ORs^25^. However other analyses found noticeable overlap between zones for rodent ORs^26,27^. Another analysis using a much larger array of mouse ORs found no clear borders between three of these expression zones, and instead a gradual shift of the midpoint of individual OR distributions^10^. A more recent analysis defines nine zones, but also mentions differences within zones^28^. Differences in morphology between fish and mammalian olfactory organ notwithstanding, the parameter analysed by Miyamichi et al.^10^ corresponds best to the radial parameter we used, since both refer to length along the sensory surface. This result, together with similar observations for laminar height (our second parameter) for rat ORs^8^, rat V2Rs^9^, frog V2Rs^11^ and frog TAARs^29^ would be consistent with the assumption that gradual changes in the distribution midpoint (one dimension examined) respectively the center-of-gravity (for more than one dimension analysed) may be the norm for different olfactory receptor genes and families in species as far apart evolutionarily as zebrafish and mice (435 mya^14^).

Remarkably these expression patterns are stable despite the limited life span of mature OSN and their ongoing renewal from stem cells^26^. Even after lesion-induced death of all mature olfactory sensory neurons these patterns can be re-established during regeneration in rats^27^. It is noteworthy that similar spatial patterns exist both for chemosensory tissues which get replenished (partially) from their borders—vomeronasal organ of mammals^30^ and olfactory epithelium of zebrafish^31,32^—and for chemosensory tissues in which OSNs are derived from basal stem cells along the sensory epithelium (rodent olfactory epithelium^33^). For rodents positional cues seem to exist^34^. For zebrafish it has been proposed that different expression zones result largely from spatial bias in OSN neurogenesis, differences in migration rates and life span of OSNs^32^. The strong intermixing of OSNs expressing different receptors, inherent in the above described spatial patterns makes it difficult to imagine how differences in center-of-gravity could enable differential functions of the respective OSNs on the level of the olfactory epithelium. Alternatively such spatial patterns could be a byproduct of other processes. Both the migration along the central-peripheral (radial) and the basal–apical (laminar height) dimension are expected to depend on OSN cell surface molecules, and in fact, OSNs expressing different receptors display major differences in the expression of cell surface guidance molecules^35^. Thus, conceivably, the epithelial sorting might be an epiphenomenon of the differential expression of surface molecules eventually needed for differential guidance of OSN axons to the olfactory bulb, where a receptotopic map is established. In such a case the spatial segregation of OSN somata described here would be a byproduct of axonal sorting and by itself without functional importance for sensing. Our in depth analysis of the spatial expression patterns for an entire olfactory receptor family will provide a basis for further mechanistic analyses of these hypotheses.

Taken together we have described broadly overlapping, but distinctly different expression zones for different ORA receptors spanning nearly the full range of the sensory surface. These expression zones intercalate with those of OR, V2R/OlfC, and AdorB receptors^7,12,17^ This spatial logic thus constitutes a general feature shared by zebrafish ORA, OR, V2R/OlfC and AdorB receptor families as well as mammalian ORs and V2Rs.

## Materials and methods

### Animal and tissue handling

Animal housing and maintenance was licensed by the office for environment and consumer protection of the city of Cologne, Germany. The experimental procedures were approved by the Federal ministry for nature, environment and consumer protection of Nordrhine-Westfalia, Germany, and were in accordance with the National Institutes of Health Guide for the Care and Use of Laboratory Animals (1996 revision).

Zebrafish used in this study are of Ab/Tü genetic background and were raised in the local fish facility at 28 °C with a 14/10 photoperiod. Adult wild type zebrafish (8–10 months old) were anesthetized with MS-222 (ethyl 3-aminobenzoate, Sigma) and decapitated. Olfactory epithelia were dissected out, embedded in TissueTek O.C.T. compound (Tissue-Tek; Sakura Finetek USA), and kept frozen until use. Ten µm horizontal cryosections were thaw-mounted onto Superfrost Plus slide glasses (Fisher Scientific, Pittsburgh, PA) and fixed for 15 min in 4% paraformaldehyde.

### In situ hybridization

Probe sequences were cloned by PCR from cDNA using standard methods and confirmed by sequencing (Table 1). Digoxigenin-labeled RNA probes were obtained by in vitro transcription according to the manufacturer's instructions (Roche Biochemicals). Hybridization was carried out overnight at 60° C using standard protocols. For chromogenic detection digoxigenin was visualized by binding to alkaline phosphatase conjugated anti-digoxigenin antibody (Roche) and subsequent enzymatic reaction with nitro blue tetrazolium chloride (Roche Biochemicals) and 5′-bromo-4-chloro-3-indolyl phosphate for 3 h at room temperature. For fluorogenic detection standard TSA method was employed (anti-digoxigenin-peroxidase, diluted 1:300 for 2 h, biotin-tyramide, Alexa Fluor488-streptavidin) and the standard protocol was modified as follows: Before acetylation the slides were incubated in 1% H_2_O_2_ for 10 min at room temperature. The blocking solution was made using TN buffer, and washing buffer was TNT buffer. Sections were mounted with Vectashield.

**Table 1:** Primer sequences.

Gene	Forward primer	Reverse primer	bp
*ora1*	ATGGACCTGTGTGTCACCATCAAAGGCGT	TCATGGAAGTCCACATGGCAGAAG	524
*ora2*	ATGATTGCGGAGGCTGTG	TCCACGTTGATGGCGTTC	495
*ora3a*	CCTCAAAAGAAACCCGTGAA	CACCATCAGGACTGTGTTCCT	234
*ora3b*	TACTACGGCAAAACCCCTGA	AATCACCAGAAGAGAGTTTCTCG	235
*ora4*	ATGTCTGAGGTCCTGACGGTG	GTGGTGCAGCTAATCACCATC	512
*ora5*	TTCTGCGTTACAGGCATCAC	AAGAAGTGAGGGACGCTGAA	397
*ora6*	GTGATGGAGCAGATACAGGAG	TCCAGTGTGTTAACCAGGAG	454

Primer sequences are given in 5′–3′ direction, size of resulting fragment is given in base pairs (bp). Primer sequences for *or* genes were as described^7^.

### Measurement and analysis of spatial coordinates

The distribution of labeled receptor neurons was assessed in complete series of olfactory epithelial sections as described previously^12^. In short, we evaluated three spatial coordinates: radial distance (center of the lamella to cell position), height within the lamella or laminar height (basal border of the lamella to cell position), and height-within-the-organ or z-axis (number of tissue section from bottom to top), see Fig. 2 for visualisation of parameters. Spatial coordinates were normalized to the respective maximal values. Distributions were visualized in three different ways: as histograms (10 bins, bin center is shown, only done for ORAs), as empirical cumulative distribution function (ECDF), i.e., unbinned^36,37^, and as two-dimensional ovoids with center at the median value and diameter representing the difference between 1st and 3rd quartile (half-width) for each dimension. Significance of differences between distributions was evaluated with Kolmogorov–Smirnov test. The KS test was performed using R^38^. This test makes no assumptions about the nature of the distributions investigated, which is essential since the skewness of many distributions shows that these are not Gaussian. Due to the sensitive nature of the test on large distributions (n > 100) we selected *p* < 0.01 as cutoff criterion for significant difference, cf.^11^ and *p* < 0.001 for very large distributions (n > 500).

### Ethics statement

All experimental procedures were carried out in compliance with local safety regulations and applicable ARRIVE guidelines.

## Supplementary Information

Supplementary Information.

## Data Availability

All data generated or analysed during this study are included in this published article and its supplementary information files.

## References

[1] Niimura Y, Matsui A, Touhara K (2014). Extreme expansion of the olfactory receptor gene repertoire in African elephants and evolutionary dynamics of orthologous gene groups in 13 placental mammals. Genome Res..

[2] Korsching SI, Fritzsch B, Meyerhof W (2020). Taste and smell in Zebrafish. The Senses: A Comprehensive Reference.

[3] Saraiva LR, Korsching SI (2007). A novel olfactory receptor gene family in teleost fish. Genome Res..

[4] Zapilko V, Korsching SI (2016). Tetrapod V1R-like ora genes in an early-diverging ray-finned fish species: The canonical six ora gene repertoire of teleost fish resulted from gene loss in a larger ancestral repertoire. BMC Genomics.

[5] Mombaerts P (2004). Odorant receptor gene choice in olfactory sensory neurons: The one receptor-one neuron hypothesis revisited. Curr. Opin. Neurobiol..

[6] Strotmann J, Wanner I, Helfrich T, Beck A, Breer H (1994). Rostro-caudal patterning of receptor-expressing olfactory neurones in the rat nasal cavity. Cell Tissue Res..

[7] Weth F, Nadler W, Korsching S (1996). Nested expression domains for odorant receptors in zebrafish olfactory epithelium. Proc. Natl. Acad. Sci. USA.

[8] Strotmann J, Konzel-mann S, Breer H (1996). Laminar segregation of odorant receptor expression in the olfactory epithelium. Cell Tissue Res..

[9] Herrada G, Dulac C (1997). A novel family of putative pheromone receptors in mammals with a topographically organized and sexually dimorphic distribution. Cell.

[10] Miyamichi K, Serizawa S, Kimura HM, Sakano H (2005). Continuous and overlapping expression domains of odorant receptor genes in the olfactory epithelium determine the dorsal/ventral positioning of glomeruli in the olfactory bulb. J. Neurosci..

[11] Syed AS, Sansone A, Nadler W, Manzini I, Korsching SI (2013). Ancestral amphibian v2rs are expressed in the main olfactory epithelium. Proc. Natl. Acad. Sci. USA.

[12] Ahuja G, Reichel V, Kowatschew D, Syed AS, Kotagiri AS, Oka Y, Weth F, Korsching SI (2018). Overlapping but distinct topology for zebrafish V2R-like olfactory receptors reminiscent of odorant receptor spatial expression zones. BMC Genomics.

[13] Ahuja G, Nia SB, Zapilko V, Shiriagin V, Kowatschew D, Oka Y, Korsching SI (2014). Kappe neurons, a novel population of olfactory sensory neurons. Sci. Rep..

[14] Kumar S, Stecher G, Suleski M, Hedges SB (2017). TimeTree: A resource for timelines, timetrees, and divergence times. Mol. Biol. Evol..

[15] Kowatschew D, Korsching SI (2022). Lamprey possess both V1R and V2R olfactory receptors, but only V1Rs are expressed in olfactory sensory neurons. Chem. Senses.

[16] Weth F (2001). Molekulare Aspekte der funktionellen Architektur des Geruchssystems beim Zebrabärbling, Danio rerio.

[17] Kowatschew D, Korsching SI (2021). An ancient adenosine receptor gains olfactory function in bony vertebrates. Genome Biol. Evol..

[18] Wakisaka N, Miyasaka N, Koide T, Masuda M, Hiraki-Kajiyama T, Yoshihara Y (2017). An adenosine receptor for olfaction in fish. Curr. Biol..

[19] Oka Y, Saraiva LR, Korsching SI (2012). Crypt neurons express a single V1R-related ora gene. Chem. Senses.

[20] Catania S, Germanà A, Laurà R, Gonzalez-Martinez T, Ciriaco E, Vega JA (2003). The crypt neurons in the olfactory epithelium of the adult zebrafish express TrkA-like immunoreactivity. Neurosci. Lett..

[21] Ahuja G, Ivandic I, Saltürk M, Oka Y, Nadler W, Korsching SI (2013). Zebrafish crypt neurons project to a single, identified mediodorsal glomerulus. Sci. Rep..

[22] Sato Y, Miyasaka N, Yoshihara Y (2005). Mutually exclusive glomerular innervation by two distinct types of olfactory sensory neurons revealed in transgenic zebrafish. J. Neurosci..

[23] Ressler KJ, Sullivan SL, Buck LB (1993). A zonal organization of odorant receptor gene expression in the olfactory epithelium. Cell.

[24] Vassar R, Ngai J, Axel R (1993). Spatial segregation of odorant receptor expression in the mammalian olfactory epithelium. Cell.

[25] Horowitz LF, Saraiva LR, Kuang D, Yoon K, Buck LB (2014). Olfactory receptor patterning in a higher primate. J. Neurosci..

[26] Norlin M, Alenius M, Gussing F, Hägglund M, Vedin V, Bohm S (2001). Evidence for gradients of gene expression correlating with zonal topography of the olfactory sensory map. Mol. Cell. Neurosci..

[27] Iwema CL, Fang H, Kurtz DB, Youngentob SL, Schwob JE (2004). Odorant receptor expression patterns are restored in lesion-recovered rat olfactory epithelium. J. Neurosci..

[28] Zapiec B, Mombaerts P (2020). The zonal organization of odorant receptor gene choice in the main olfactory epithelium of the mouse. Cell Rep..

[29] Syed AS, Sansone A, Röner S, Nia SB, Manzini I, Korsching SI (2015). Different expression domains for two closely related amphibian TAARs generate a bimodal distribution similar to neuronal responses to amine odors. Sci. Rep..

[30] Martínez-Marcos A, Ubeda-Bañón I, Deng L, Halpern M (2000). Neurogenesis in the vomeronasal epithelium of adult rats: Evidence for different mechanisms for growth and neuronal turnover. J. Neurobiol..

[31] Oehlmann VD, Berger S, Sterner C, Korsching SI (2004). Zebrafish beta tubulin 1 expression is limited to the nervous system throughout development, and in the adult brain is restricted to a subset of proliferative regions. Gene Expr. Patterns.

[32] Bayramli X, Kocagöz Y, Sakizli U, Fuss SH (2017). Patterned arrangements of olfactory receptor gene expression in zebrafish are established by radial movement of specified olfactory sensory neurons. Sci. Rep..

[33] Calof AL, Mumm JS, Rim PC, Shou J (1998). The neuronal stem cell of the olfactory epithelium. J. Neurobiol..

[34] Coleman JH, Lin B, Louie JD, Peterson J, Lane RP, Schwob JE (2019). Spatial determination of neuronal diversification in the olfactory epithelium. J. Neurosci..

[35] Wang I-H (2022). Spatial transcriptomic reconstruction of the mouse olfactory glomerular map suggests principles of odor processing. Nat. Neurosci..

[36] Feller W (1967). An Introduction to Probability Theory and Its Applications.

[37] Wilk MB, Gnanadesikan R (1968). Probability plotting methods for the analysis of data. Biometrika.

[38] R-core-team (2013). A Language and Environment for Statistical Computing.

