# Supplementary material for: Spatial organization of olfactory receptor gene choice in the complete V1R-related ORA family of zebrafish
Source: Sci Rep. 2022 Aug 31;12:14816. doi: 10.1038/s41598-022-17900-x (PMC9433392; doi:10.1038/s41598-022-17900-x)
Supplement: Supplementary file 1 — Supplementary Information. [file 41598_2022_17900_MOESM1_ESM.pdf]

Spatial organization of olfactory receptor gene choice in the complete V1R-related ORA family of zebrafish

Daniel Kowatschew, Shahrzad Bozorg Nia, Shahzaib Hassan,
Jana Ustinova, Franco Weth, Sigrun I. Korsching

SI Figure 1: TSA-labeling for the entire ORA family and comparison of labeling efficacy for TSA and chromogenic labeling methods for in situ hybridisation

Horizontal sections of adult zebrafish olfactory epithelium were hybridized with probes for ORA1, ORA2, ORA3a, ORA3b, ORA4, ORA5 and ORA6. Note that for better visualization sections with above average frequency are shown. Bottom right panel, expression frequencies for different labeling methods are compared. Missing bars, numbers too small for accurate determination. Mean values and SEM are shown; black bars, chromogenic labeling with NBT/BCIP (only for ORA1, ORA2, ORA3a, ORA4); white bars, TSA labeling (all 7 genes); grey bars, numbers/section obtained for both methods were averaged. Numbers within/above bars refer to the number of sections analysed for the respective bar, in parentheses the number of organs analysed is given.

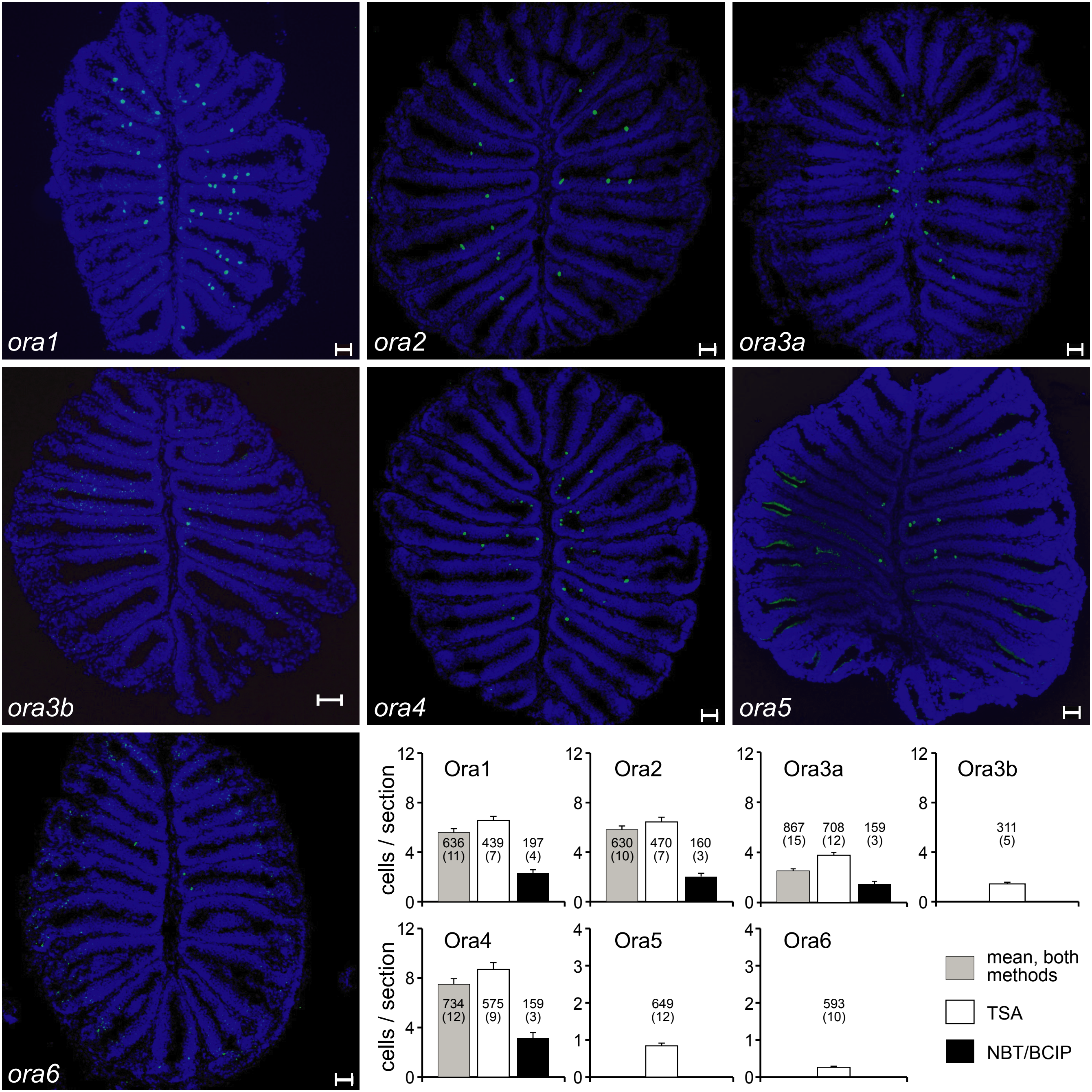

ora1
ora2
ora3a
ora3b
ora4
ora5
ora6
cells / section
Ora1
636 (11)
439 (7)
197 (4)
Ora2
630 (10)
470 (7)
160 (3)
Ora3a
867 (15)
708 (12)
159 (3)
Ora3b
311 (5)
Ora4
734 (12)
575 (9)
159 (3)
Ora5
649 (12)
Ora6
593 (10)
mean, both methods
TSA
NBT/BCIP

Spatial organization of olfactory receptor gene choice in the complete V1R-related ORA family of zebrafish

Daniel Kowatschew, Shahrzad Bozorg Nia, Shahzaib Hassan,
Jana Ustinova, Franco Weth, Sigrun I. Korsching

SI Figure 2: Multidimensional analysis of spatial expression patterns: No correlation between preferred positions for height-within-the-organ and the other two parameters, radius and laminar height, but clear segregation from two other olfactory receptor families for height-within-the-organ

A) Comparison of spatial distribution parameters between *ora* genes. Schematic representation of spatial distributions for all *ora* genes by ellipses ranging from the 1st to the 3rd quartile value for height-within-the-organ (y axis) and radial or laminar height (x axis) parameter. Color code for *ora* genes as indicated.

B) Comparison of distributions for *ora* versus *olfC* and odorant receptor (*or*) genes using the same coordinates and representation as in A). Note that the color code is changed compared to panel A). The raw data for *olfC* genes are taken from [11], raw data for *or* genes are from [7] [16]. For names and quartile values of *or* and *olfC* genes see SI Table 1.

C) Distributions of *ora*-expressing OSNs for height-within-the-organ segregate from those of other olfactory receptor families despite broad overlap. Distributions are shown as ECDFs. Red lines, *ora* genes; black, *olfC* genes; blue, *or* genes.

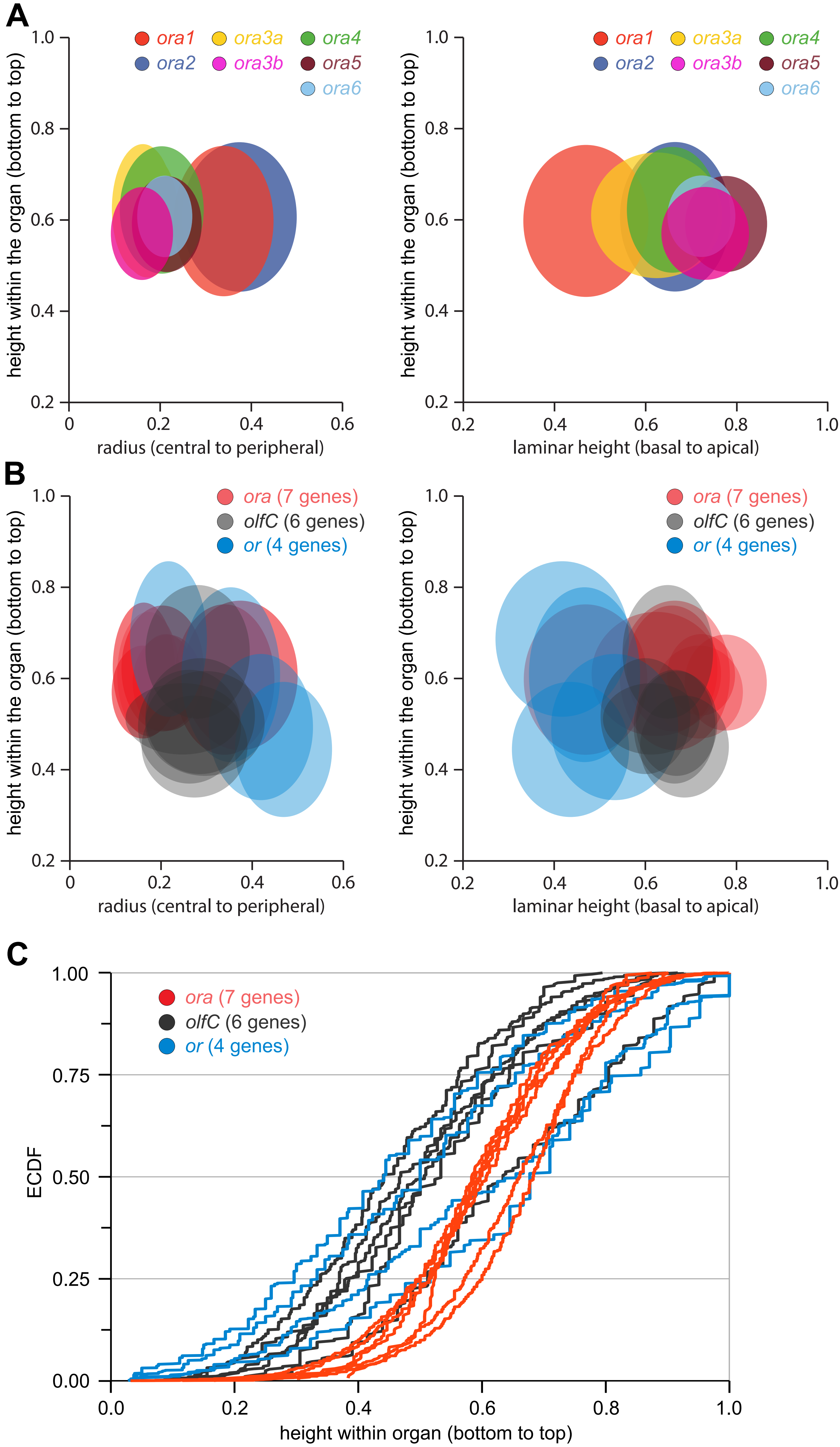

A
ora1
ora2
ora3a
ora3b
ora4
ora5
ora6
height within the organ (bottom to top)
radius (central to peripheral)
laminar height (basal to apical)
B
ora (7 genes)
olfC (6 genes)
or (4 genes)
height within the organ (bottom to top)
radius (central to peripheral)
laminar height (basal to apical)
C
ora (7 genes)
olfC (6 genes)
or (4 genes)
ECDF
height within organ (bottom to top)

Spatial organization of olfactory receptor gene choice in the complete V1R-related ORA family of zebrafish

Daniel Kowatschew, Shahrzad Bozorg Nia, Shahzaib Hassan,
Jana Ustinova, Franco Weth, Sigrun I. Korsching

SI Figure 3: Origin of the ORA3 duplication in the MRCA of cypriniformes

A) Taxonomic tree of Cypriniformes redrawn from [Stout et al., 2016]. Only those families and larger groups are included, for which genomes were available, color code as indicated. Two adjacent orders (Characiformes and Siluriformes) are also shown. B) Maximum likelihood tree of the entire ORA family (amino acid sequences) from four reference species - two early ray-finned species (*Acipenser ruthenus* and *Lepisosteus oculatus*), zebrafish and a neoteleost (medaka) - and all validated Cypriniformes, Characiformes and Siluriformes sequences of the ORA3,4 clade obtained from tblastn searches in the WGS of the respective orders. Search strategy and tree construction as described in [17]. The rainbow-colored triangle corresponds to the subtree shown in C. C) Maximum likelihood tree showing all *ora3* genes of Cypriniformes, Characiformes (collapsed) and Siluriformes (collapsed). Color code for fish families and larger groups as indicated. Zebrafish ORA3a and ORA3b are indicated by red arrows. Two separate clades for ORA3a and ORA3b include already the earliest deriving Cypriniformes (sucker and loaches), suggesting an origin of this duplication in the MRCA of Cypriniformes.

Supplementary Reference

Stout, C. C., Tan, M., Lemmon, A. R., Lemmon, E. M. & Armbruster, J. W. Resolving Cypriniformes relationships using an anchored enrichment approach. *BMC Evol Biol* **16**, 244 (2016).

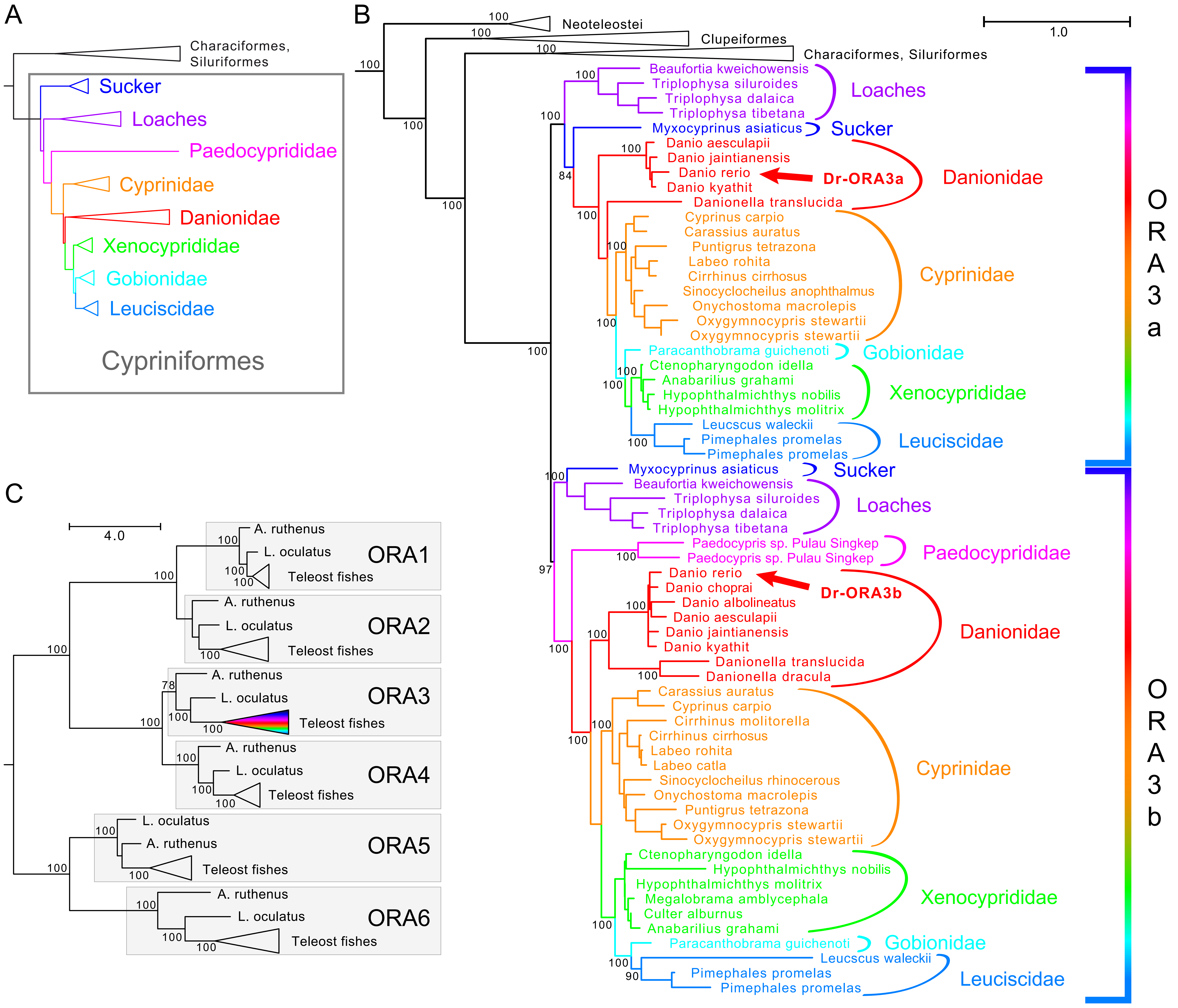

A
Characiformes, Siluriformes
Sucker
Loaches
Paedocyprididae
Cyprinidae
Danionidae
Xenocyprididae
Gobionidae
Leuciscidae
Cypriniformes
B
Neoteleostei
Clupeiformes
Characiformes, Siluriformes
1.0
Beaufortia kweichowensis
Triplophysa siluroides
Triplophysa dalaica
Triplophysa tibetana
Loaches
Myxocyprinus asiaticus
Sucker
Danio aesculapii
Danio jaintianensis
Danio rerio
Danio kyathit
Dr-ORA3a
Danionella translucida
Danionidae
Cyprinus carpio
Carassius auratus
Puntigrus tetrazona
Labeo rohita
Cirrhinus cirrhosus
Sinocyclocheilus anophthalmus
Onychostoma macrolepis
Oxygymnocypris stewartii
Oxygymnocypris stewartii
Cyprinidae
Paracanthobrama guichenoti
Gobionidae
Ctenopharyngodon idella
Anabarilius grahami
Hypophthalmichthys nobilis
Hypophthalmichthys molitrix
Xenocyprididae
Leucscus waleckii
Pimephales promelas
Pimephales promelas
Leuciscidae
ORA3a
Myxocyprinus asiaticus
Sucker
Beaufortia kweichowensis
Triplophysa siluroides
Triplophysa dalaica
Triplophysa tibetana
Loaches
Paedocypris sp. Pulau Singkep
Paedocypris sp. Pulau Singkep
Paedocyprididae
Danio rerio
Danio choprai
Danio albolineatus
Danio aesculapii
Danio jaintianensis
Danio kyathit
Dr-ORA3b
Danionella translucida
Danionella dracula
Danionidae
Carassius auratus
Cyprinus carpio
Cirrhinus molitorella
Cirrhinus cirrhosus
Labeo rohita
Labeo catla
Sinocyclocheilus rhinocerous
Onychostoma macrolepis
Puntigrus tetrazona
Oxygymnocypris stewartii
Oxygymnocypris stewartii
Cyprinidae
Ctenopharyngodon idella
Hypophthalmichthys nobilis
Hypophthalmichthys molitrix
Megalobrama amblycephala
Culter alburnus
Anabarilius grahami
Xenocyprididae
Paracanthobrama guichenoti
Gobionidae
Leucscus waleckii
Pimephales promelas
Pimephales promelas
Leuciscidae
ORA3b
C
4.0
A. ruthenus
L. oculatus
Teleost fishes
ORA1
ORA2
ORA3
ORA4
ORA5
ORA6

Spatial organization of olfactory receptor gene choice in the complete V1R-related ORA family of zebrafish

Daniel Kowatschew, Shahrzad Bozorg Nia, Shahzaib Hassan,
Jana Ustinova, Franco Weth, Sigrun I. Korsching

SI Table 1 Quantitative parameters of distributions for all ora *genes*

Sheet 1, Accession numbers and synonyms for all zebrafish *ora* genes and those *or* and *olfC* genes used in this study. Sheet 2, Parameter values for the first, second and third quartile of the distributions, i.e., for the radial, laminar height and z-axis coordinates. Half width of distributions was determined as 3rd quartile-1st quartile difference. Sheet 3, Maximal vertical distance in pairwise comparisons for all seven *ora* genes, for all four *or* genes and for ora genes with similar distribution to genes from other families in at least one dimension. Sheet 4, Evaluation of significant differences by KS test for pairwise comparisons.

gene	Synonyms	Ensembl Gene ID	NCBI
ora1	V1R2, V1r-like, vn1r1l, vn1r2l, zgc:19431	ENSDARG00000098601	NM_001130668.1
ora2	V1R1	not annotated	NM_001098395.1
ora3a	ora3, V1R3	ENSDARG00000059315	XM_009295769.2
ora3b	-	ENSDARG00000091638	XM_009295770.3
ora4	V1R4	ENSDARG00000078223	XM_005168282.4
ora5	V1R5	ENSDARG00000078257	XM_002663460.5
ora6	V1R6	not annotated	XM_003200787.2
olfcc1	v2rl1	ENSDARG00000005942	XM_009293770
olfcg1		ENSDARG00000104552	NM_001159840
olfcn1	si:ch73-125k17.2	ENSDARG00000088029	XM_009293708, XM_009293709
olfcq1	v2rh14	ENSDARG00000068520	NM_001083099
olfcj1		ENSDARG00000104430	NM_001110291
olfct1		not present in z10	XM_021473301
olfcu1	v2rx1	ENSDARG00000040632	NM_001082899
or102-1	ZOR5	ENSDARG00000041005	XM_009291682
or112-1	ZOR6	ENSDARG00000077211	XM_003200004
or103-1	ZOR8	ENSDARG00000094080	NM_131588
or107-1	ZOR9	ENSDARG00000041032	XM_009291685

Expression of V1R-related *ora* genes in zebrafish olfactory epithelium: Distribution of three coordinates, radius, laminar height, and height within the organ

Radial coordinate

Half width := 3rd -1st quartile

Values are normalized radial length (r/ro)

receptor	1st quartile	2st quartile	3rd quartile	half width
ora1	0,230	0.340	0.449	0.219
ora2	0.248	0.376	0.500	0.251
ora3a	0.094	0.147	0.230	0.136
ora3b	0.092	0.143	0.228	0.136
ora4	0.113	0.192	0.294	0.181
ora5	0.138	0.204	0.291	0.153
ora6	0.150	0.223	0.270	0.121
zor5	0.362	0.494	0.575	0.213
zor6	0.132	0.198	0.301	0.169
zor8	0.305	0.436	0.533	0.229
zor9	0.245	0.363	0.461	0.216
olfcc1	0.233	0.342	0.458	0.225
olfcg1	0.157	0.262	0.389	0.232
olfcj1	0.122	0.210	0.372	0.250
olfcn1	0.149	0.247	0.375	0.226
olfcq1	0.165	0.283	0.427	0.262
olfct1	0.166	0.299	0.406	0.240
olfcu1	0.154	0.259	0.382	0.229

Laminar height coordinate

Values are normalized laminar length (h/ho)

Gene	1st quartile	2st quartile	3rd quartile	half width
ora1	0.333	0.469	0.604	0.271
ora2	0.544	0.672	0.785	0.242
ora3a	0.482	0.650	0.766	0.284
ora3b	0.634	0.747	0.826	0.192
ora4	0.559	0.671	0.758	0.199
ora5	0.687	0.808	0.866	0.180
ora6	0.648	0.722	0.797	0.149
zor5	0.307	0.436	0.564	0.257
zor6	0.271	0.395	0.564	0.293
zor8	0.393	0.529	0.672	0.279
zor9	0.345	0.457	0.588	0.244
olfcc1	0.541	0.658	0.757	0.216
olfcg1	0.588	0.714	0.783	0.195
olfcj1	0.507	0.603	0.719	0.212
olfcn1	0.575	0.678	0.754	0.179
olfcq1	0.579	0.676	0.758	0.178
olfct1	0.502	0.615	0.699	0.197
olfcu1	0.550	0.647	0.749	0.199

Z-axis coordinate

Values are normalized height within organ (section no/total # sections)

Gene	1st quartile	2st quartile	3rd quartile	half width
ora1	0.500	0,607	0.705	0.205
ora2	0.500	0.589	0.689	0.189
ora3a	0.571	0.661	0.750	0.179
ora3b	0.515	0.597	0.677	0.163
ora4	0.600	0.683	0.746	0.146
ora5	0.518	0.599	0.705	0.187
ora6	0.525	0.584	0.689	0.164
zor5	0.296	0.441	0.593	0.296
zor6	0.516	0.677	0.857	0.341
zor8	0.333	0.500	0.654	0.321
zor9	0.417	0.655	0.800	0.383
olfcg1	0.339	0.441	0.563	0.224
olfcj1	0.433	0.500	0.590	0.156
olfcn1	0.370	0.469	0.619	0.249
olfcq1	0.387	0.507	0.616	0.230
olfct1	0.390	0.533	0.644	0.254
olfcu1	0.512	0.634	0.805	0.293

Pairwise comparison of spatial patterns for expression of different *ora* genes in zebrafish olfactory epithelium: maximal vertical distance between distributions (determined in Kolmogorov-Smirnov test)

Three coordinates were evaluated: radius, laminar height, and height within the organ (z-axis)

		Maximal vertical distance between the two distributions compared		
**Gene 1**	**Gene 2**	**Radius**	**Laminar Height**	**Z-axis**
		**r/ro**	**h/ho**	**z/zo**
ora1	*ora2*	0.110	0.409	0.065
ora1	*ora3a*	0.516	0.404	0.174
ora1	*ora3b*	0.512	0.404	0.085
ora1	*ora4*	0.371	0.432	0.248
ora1	*ora5*	0.377	0.628	0.065
ora1	*ora6*	0.430	0.558	0.137
ora2	*ora3a*	0.550	0.057	0.219
ora2	*ora3b*	0.550	0.207	0.066
ora2	*ora4*	0.422	0.081	0.286
ora2	*ora5*	0.436	0.323	0.067
ora2	*ora6*	0.490	0.205	0.142
ora3a	*ora3b*	0.057	0.206	0.226
ora3a	*ora4*	0.197	0.077	0.099
ora3a	*ora5*	0.267	0.354	0.197
ora3a	*ora6*	0.313	0.201	0.240
ora3b	*ora4*	0.189	0.236	0.311
ora3b	*ora5*	0.257	0.195	0.063
ora3b	*ora6*	0.305	0.126	0.125
ora4	*ora5*	0.120	0.400	0.275
ora4	*ora6*	0.146	0.200	0.302
ora5	*ora6*	0.075	0.286	0.104

zor5	*zor6*	0.602	0.087	0.438
zor5	*zor8*	0.162	0.196	0.132
zor5	*zor9*	0.347	0.084	0.324
zor6	*zor8*	0.523	0.268	0.366
zor6	*zor9*	0.399	0.152	0.148
zor8	*zor9*	0.194	0.158	0.259

zor5	*ora1*	0.374	0.074	0.276
zor6	*ora1*	0.359	0.141	0.221
zor8	*ora1*	0.232	0.140	0.186
zor9	*ora1*	0.078	0.049	0.100
olfcc1	*ora1*	0.028	0.402	n.d.
olfcn1	*ora2*	0.267	0.104	0.250
olfcq1	*ora2*	0.210	0.100	0.250
olfcn1	*ora4*	0.191	0.047	0.334
olfcq1	*ora4*	0.244	0.070	0.320
olfcu1	*ora4*	0.181	0.071	0.080

Expression patterns of V1R-related *ora* genes in zebrafish olfactory epithelium:
Distribution of radial coordinate, laminar height, and height within organ (z axis)

Pairwise comparison of distributions: p-value determined by Kolmogorov-Smirnov test.
Significant p values are overlayed grey (p<0.01 is considered significant for 100<n<500 and p<0.001 for n>500).

Gene 1	Gene 2	p value Radius	p value Laminar Height	p value Z-axis
ora1	*ora2*	4.90E-011	2.20E-016	7.81E-007
ora1	*ora3a*	2.20E-016	2.20E-016	2.20E-016
ora1	*ora3b*	2.20E-016	2.20E-016	0.006393
ora1	*ora4*	2.20E-016	2.20E-016	2.20E-016
ora1	*ora5*	2.20E-016	2.20E-016	0.1191
ora1	*ora6*	2.20E-016	2.20E-016	0.007076
ora2	*ora3a*	2.20E-016	0.013	2.20E-016
ora2	*ora3b*	2.20E-016	1.40E-009	0.0669
ora2	*ora4*	2.20E-016	1.63E-007	2.20E-016
ora2	*ora5*	2.20E-016	2.20E-016	0.09496
ora2	*ora6*	2.20E-016	6.90E-005	0.004451
ora3a	*ora3b*	0.475	1.40E-008	2.20E-016
ora3a	*ora4*	2.20E-016	1.00E-004	3.33E-016
ora3a	*ora5*	2.20E-016	2.20E-016	2.06E-011
ora3a	*ora6*	2.60E-010	2.00E-004	6.47E-008
ora3b	*ora4*	3.50E-008	1.75E-012	2.20E-016
ora3b	*ora5*	4.30E-009	1.90E-005	0.398
ora3b	*ora6*	1.60E-007	0.120	0.05134
ora4	*ora5*	0.0003352	2.20E-016	2.20E-016
ora4	*ora6*	0.1076	1.07E-004	1.38E-012
ora5	*ora6*	0.6647	4.50E-007	0.181

zor5	*zor6*	2.20E-016	0.09894	2.20E-016
zor5	*zor8*	1.35E-006	2.20E-016	3.24E-012
zor5	*zor9*	2.20E-016	0.00107	2.20E-016
zor6	*zor8*	2.20E-016	5.38E-010	2.20E-016
zor6	*zor9*	2.20E-016	0.0002161	2.77E-006
zor8	*zor9*	1.64E-011	9.50E-013	2.20E-016

zor5	*ora1*	2.20E-016	0.001342	2.20E-016
zor6	*ora1*	2.20E-016	0.000423	8.37E-009
zor8	*ora1*	2.20E-016	7.26E-013	2.53E-009
zor9	*ora1*	0.001517	0.08032	0.007384
olfcc1	*ora1*	0.9721	6.16E-013	n.d.
olfcn1	*ora2*	5.55E-016	0.002907	2.63E-011
olfcq1	*ora2*	2.49E-014	0.002541	2.30E-013
olfcn1	*ora4*	1.88E-008	0.5118	2.20E-016
olfcq1	*ora4*	2.20E-016	0.07049	2.20E-016
olfcu1	*ora4*	2.64E-007	0.1839	0.2301
